# Endometriosis accelerates synchronization of early embryo cell divisions but does not change morphokinetic dynamics in endometriosis patients

**DOI:** 10.1371/journal.pone.0220529

**Published:** 2019-08-01

**Authors:** Michael Schenk, Julia Maria Kröpfl, Martina Hörmann-Kröpfl, Gregor Weiss

**Affiliations:** 1 Das Kinderwunsch Institut Schenk GmbH, Dobl, Austria; 2 Institute of Human Genetics, Medical University of Graz, Graz, Austria; 3 Exercise Physiology Lab, Institute of Human Movement Sciences and Sport, ETH Zurich, Zurich, Switzerland; Hull York Medical School, UNITED KINGDOM

## Abstract

**Objective:**

The pathology of endometriosis and its impact on embryo development is still a black box in reproductive medicine. In this time-lapse study we investigated the influence of endometriosis on morphokinetic parameters of embryo development, taking variables of dynamic monitoring into account. Furthermore we evaluated reproductive medicine treatment outcome such as fetal heartbeat and live birth rate.

**Methods:**

1148 embryos (control: n = 596, endometriosis: n = 552) were retrospectively analyzed. Patients were stimulated with GnRH antagonist protocol. After fertilization, embryos were incubated in a time-lapse system (EmbryoScope).

**Results:**

The mixed-model analysis revealed a significant main effect of time (p<0.001), with post-hoc tests showing that any time needed to reach a specific developmental stage was significantly different from all the others (all p<0.001). Embryos of endometriosis patients showed the same absolute morphokinetic time parameters as the control group, however, synchronization of early embryo cell divisions (s2) was faster in endometriosis patients compared to the control group.

**Conclusion:**

In general, endometriosis does not induce changes in early embryo morphokinetics. However, observed acceleration in cell cycle synchronization of embryo cleavage patterns might be a missing explanation for contradicting results in literature regarding the impairments in reproductive medicine treatment outcome of endometriosis patients.

## Introduction

Endometriosis is defined as the presence of endometrial tissue outside the uterine cavity and is one of the leading gynecologic disorders affecting approximately 10% of women in reproductive age [1]. Despite its high prevalence, the pathology and mechanisms of how endometriosis impairs female fertility remain largely unknown. Many symptoms are closely related to other disorders and the availability of concise biomarkers in blood or urine is limited [2,3]. Endometriosis is frequently associated with impaired fertilization and implantation characterized by abnormal folliculogenesis, elevated oxidative stress, altered immune function and hormonal milieu in the follicular and peritoneal environments, and reduced endometrial receptivity [4]. Furthermore, higher spindle abnormalities in oocytes from endometriosis patients have been reported [5]. Severe stages of endometriosis might also lead to deteriorating mechanical disturbances of the female reproductive system [6].

The most effective method to overcome endometriosis-related infertility is represented by assisted reproductive technology (ART) *in vitro* fertilization (IVF) or intracytoplasmic sperm injection (ICSI). However, whether endometriosis affects ART-outcome is still controversially discussed. Recent studies assume a similar ART-outcome of patients with and without endometriosis, however, there is a higher risk of miscarriage in endometriosis patients [7]. Additionally, the number of retrieved oocytes is reduced in advanced stages of the disease and lower oocyte quality has been observed in some studies [7,8]. As a result, it is tempting to speculate that the severity of the disease might also have an impact on embryo development in regards of morphokinetic timings. A better characterization of endometriosis’ pathology by comparing morphokinetic cell cycle durations and the synchronization of cell divisions between all stages of the disease has not yet been performed.

In the last years, time-lapse technology has evolved to become a potent tool to track and study embryo morphokinetics. The success is based on the minimization of environmental influences [9,10], while enabling the observation of embryo development. A recent Cochrane review stated that there is insufficient evidence of differences in live birth, or clinical pregnancy between time-lapse and conventional incubation [11]. However, additional information on kinetic markers have been proven excellent tools to evaluate distortions in cell cleavage times of the embryo [12,13]. Furthermore, the visualization of abnormal embryo development patterns like direct or reverse cleavage is an additional criterion commonly used in embryo selection [14].

To date, the influence of endometriosis on early embryo morphokinetics has only been investigated by two studies [15,16]. The authors observed altered relative kinetics in embryos from patients with endometriosis compared to controls, but results disagree regarding differences in variables of dynamic monitoring. An impact of endometriosis on early embryo morphokinetics has been suggested [16] but the incomplete statistical analysis used is very likely to have biased found results.

Hence, in the present study we wanted to verify and extend current knowledge by investigating the influence of endometriosis on morphokinetic parameters of early embryo development using time-lapse technology. We hypothesized that alterations in absolute or dynamic monitoring variables may be reasons for impairments of outcome in ART-treatment of endometriosis patients also taking into consideration the different stages of endometriosis and the day of embryo transfer.

## Material and methods

### Patients’ characteristics

In total, 1148 embryos (endometriosis group: n = 552, control group: n = 596) from 77 controls and 86 patients (age 26–39) with endometriosis undergoing ICSI treatment were included in the study. Patients who met the following criteria were excluded: [a] obesity (BMI > 30), [b] anorexia (BMI < 17.5), [c] age above 39 and under 26, [d] nicotine abuse, [e] endocrine disorders (including polycystic ovarian syndrome, reduced ovarian reserve, menopause, hypothalamic amenorrhea, congenital adrenal hyperplasia), [f] diabetes mellitus, [g] chronic inflammation, [h] known genetic disorders and [i], severe oligoasthenoteratozoospermia (OAT). Control patients suffered from unexplained or prolonged infertility and were matched according to age and body mass index (BMI). Endometriosis patients were further subdivided into the 4 American Socienty for Reproductive Medicine (ASRM) categories [17]: stage I (minimal): n = 38, stage II (mild): n = 21, stage III (moderate): n = 20 and stage IV (severe): n = 7.

The number of oocytes retrieved varied between 1 and 30 per patient and cycle, in total 2117 oocytes were retrieved. Divided by ASRM categories, 212 embryos (38.4%) were in the minimal, 143 (25.9%) in the mild, 154 (27.9%) in the moderate and 43 (7.8%) in the severe endometriosis group. The number of embryos (cases) varied between 1 and 30 per patient. Not all embryos progressed to the morula stage. This was taken into account during statistical analysis.

The clinical records were collected at the IVF institution “Das Kinderwunsch Institut Schenk GmbH” in Dobl, Austria, between 2014 and 2016 from patients who had signed an informed consent, in which they consented to have data from their medical records used in research. The study was approved by the ethical committee of the Medical University of Graz, Austria (approval number: 28–401 ex 15/16).

### Controlled ovarian stimulation

Ovarian hyperstimulation was performed as previously described [18]. Briefly, patients received controlled ovarian stimulation with gonadotropin-releasing hormone (GnRH) antagonist protocol. Recombinant human follicle stimulating hormone (Puregon; MSD Sharp & Dohme GMBH, Haar, Germany) was administered during 5 days, the dose was adjusted to age, weight, AMH and hormonal status [19,20]. Ovarian response was monitored using ultrasonographical measurement RIC 5-9-D 4D intravaginal probe of a GE Voluson E8 BT09 ultrasound machine (both from GE Healthcare Austria GmbH, Pfaffing, Austria). Premature ovulation was prevented with GnRH antagonist (Cetroide, Merck KGaA, Darmstadt, Germany).

### Oocyte retrieval and fertilization

Oocyte retrieval was performed using a Steiner-Tan needle 17 gauge and a Steiner flush/valve (IVFETFLEX.com HandelsgmbH & Co KG, Graz, Austria) 35 hours after administering 5,000–10,000 IU human chorionic gonadotropin (Pregnyl, Merck Sharp & Dohme Ges.m.b.H., Vienna, Austria) [19]. Oocytes were processed under constant conditions of 37°C in an IVF workstation L24E with an included heating stage (K-SYSTEMS Kivex Biotec A/S, Birkerod, Denmark) as previously described [18]. ICSI was performed in all oocytes of both groups (endometriosis and control). Insemination of M-II oocytes in all patients took place 4–5 hours after oocyte retrieval according to our standard operating procedure. All procedures were performed according to the standard protocols of collection and storage of body liquids within the frame of ART-treatment [21,22].

### Time-lapse incubation and embryo analysis

After oocyte retrieval and fertilization, oocytes were cultured in a Forma CO_2_ incubator (Thermo Fisher Scientific, Waltham, USA) with universal culture medium (Gynemed Medizinprodukte GmbH & Co.KG, Lensahn, Germany). Fertilization check was performed after a time period of 16–18 hours. Subsequently, embryos with 2 pronuclei (PN) were cultured in an Embryoscope time-lapse incubator (Vitrolife AB, Göteborg, Sweden) at stable conditions of 21% oxygen concentration, 6% CO_2_ and 37°C. Time-lapse acquisition was set at 15 minute intervals in 9 focal planes. Morphokinetic parameters and parameters of dynamic monitoring of embryo development were described according to the criteria proposed by Ciray and coworkers [23] (Table 1). Analysis was performed using a software developed for Embryoscope (Embryoviewer software; Vitrolife AB). In addition, irregular events in embryo development such as direct cleavage (a single blastomere divides directly from 1 to 3 cells in less than 5 hours) and reverse cleavage (a blastomere is re-absorbed after cleavage) were annotated according to the description by Rubio et al. [14].

**Table 1:** Morphokinetic parameters and dynamic monitoring variables and proposed definitions adapted from Ciray et al. [23].

Time	*Definition of expected events*
t0	Time of IVF or mid-time of micro/injection (ICSI)
tPN	Fertilization status is confirmed
tPNf	Time of pronuclei disappearance; tPN1f; tPN2f.
t2 to t9	Two to nine discrete cells
tMor	End of compaction process (last frame before cavity formation)
ECC2	Duration of second embryo cell cycle (t4-t2)
cc2a-b	Cell cycle for blastomere a (t3-t2), b (t4-t2)
ECC3	Duration of the third embryo cell cycle (t8-t4)
cc3a-d	Cell cycle for blastomere a (t5-t4), b (t6-t4), c (t7-t4), d (t8-t4)
s2	Synchronization of cell divisions (t4-t3)

### Embryo transfer

The day of embryo transfer was chosen for each patient taking into consideration the number of fertilized oocytes, patient age and embryo quality in previous attempts (if any), regardless of the presence of endometriosis. Day 4 embryo transfer was only performed on patients’ demand. The Istanbul consensus grading scheme for d3, d4 and d5 embryos [24] was used as morphology assessment criteria to score all embryos at the day of transfer. The best viable embryo was transferred using an embryo transfer catheter set (Labotect Labor Technik Göttingen GmbH, Goettingen, Germany). Double embryo transfer was performed on patients’ demand 15 times in the control group and 13 times in the endometriosis group. Luteal supplementation was provided by vaginal progesterone (Utrogestan, Vifor Pharma, Villars-sur-Glâne, Switzerland).

### Statistical analysis

Data are given as mean [95% confidence intercal (CI)], mean predicted values [95% CI], absolute or percent values. Depending on variable distributions tested by the Kolmogorov-Smirnov test, clinical outcome variables such as retrieved oocytes/cycle, transferred embryos/cycle and implanted embryos/cycle were compared between groups using a One-way ANOVA or Kruskal-Wallis test with post-hoc Bonferroni comparisons. Calculated variables of dynamic monitoring of embryo development, such as ECC2 (cc2a, cc2b) and ECC3 (cc3a-d), s2 (t4-t3) and s3 (t8-t5) [23] were analyzed using a linear mixed effect model (restricted maximum likelihood) with one fixed factor (endometriosis yes/no or stages of endometrionsis) and one random factor (patient ID) with Bonferroni post-hoc comparisons including age, BMI and cycle as covariates. Embryo morphokinetics were also addressed by a linear mixed effect model (restricted maximum likelihood) approach in order to properly deal with missing data, compare unequal sample sizes and account for multiple IVF cycles in the context of a scaled outcome variable [25]. The model included two fixed factors (repeated: developmental stages, group effect: endometriosis yes/no or stages of endometriosis) with Bonferroni post-hoc comparisons, a random intercept (patient ID) and a random slope as well as three covariates (age, BMI, cycle). Interaction effects were also post-hoc corrected for multiple testing (Bonferroni). The time in hours to reach a specific developmental stage, measured by time-lapse technology, was the dependent variable in the model. The model selection process to define the appropriate covariance structure of the repeated effect and the random effect was based on ACI (Akaike Information Criterion), an index of relative goodness-of-fit. Finally, an autoregressive covariance structure for the repeated, fixed effect and variance components for the random effect (random intercept and slope) were used. Since some patients were lacking a BMI value, only n = 1,007 embryos were included in the linear mixed effect model (control group: n = 542; endometriosis group: n = 465 composed of n = 171, 130, 138, 26 for minimal, mild, moderate, and severe endometriosis, respectively).

Relationships between continuous variables were evaluated by Pearson’s or Spearman’s correlation analyses depending on variables’ distributions. Correlations between continuous and nominal variables were done by (point-) biserial correlation analyses and associations between nominal variables were computed by Chi-Squared/Fisher’s exact test depending on the number of included cases. A p-value (two-tailed) of <0.05 was considered as statistically significant. Statistical analysis was performed using SPSS 23.0.0.2 (SPSS Inc., Chicago, IL) for calculations and graphs as well as GraphPad Prism 7.0a (GraphPad Software, San Diego, USA) as support for visualizations.

## Results

### Patients’ characteristics and clinical data

Patients’ characteristics and clinical data for the different stages of endometriosis are shown in Table 2. There were neither significant differences for patients’ age, BMI, retrieved oocytes/cycle, transferred embryos/cycle and implanted embryos/cycle between the endometriosis and the control group nor between the different stages of endometriosis and the control group (Table 2). The average number of cycles in the endometriosis group was comparable to the one in the control group.

**Table 2:** Patients’ characteristics and clinical data for the control and endometriosis group as well as the different stages of endometriosis.

	CONTROL GROUP	ALL ENDOMETRIOSIS	MINIMAL ENDOMETRIOSIS	MILD ENDOMETRIOSIS	MODERATE ENDOMETRIOSIS	SEVERE ENDOMETRIOSIS
***Patients’ characteristics***						
Age (years)	33.3 [33.0, 33.7]	32.1 [31.7, 32.5]	31.7 [30.9, 32.5]	33.3 [32.6, 34.1]	32.0 [31.4, 32.7]	29.8 [29.0, 30.7]
BMI (kg/m^2^)	22.6 [22.3, 23.0]	22.7 [22.4, 23.0]	23.8 [23.1, 24.5]	22.4 [21.9, 23.0]	21.7 [21.3, 22.1]	21.9 [21.0, 22.9]
***Clinical data***						
Total number of cycles	134	118	41	33	35	9
Average number of cycles	2.1 [1.9, 2.2]	1.8 [1.7, 1.9]	1.5 [1.4, 1.6]	1.7 [1.5, 1.8]	2.4 [2.1, 2.7]	1.5 [1.2, 1.8]
Retrieved oocytes /cycle	7.9 [7.1, 8.8]	8.9 [7.8, 10.1]	9.1 [6.7, 11.4]	8.9 [7.0,10.8]	9.0 [7.2, 10.8]	8.4 [1.6, 15.3]
Transferred embryos /cycle	1.5 [1.3, 1.6]	1.4 [1.3, 1.6]	1.3 [1.1, 1.6]	1.4 [1.1, 1.7]	1.6 [1.4, 1.8]	1.3 [0.8, 1.9]
Implanted embryos / cycle	0.46 [0.35, 0.57]	0.42 [0.32, 0.52]	0.49 [0.31, 0.66]	0.36 [0.17, 0.66]	0.46 [0.26, 0.65]	0.22 [0.00, 0.56]
Implantation rate / cycle (%)	31.8	29.6	36.4	25.5	29.1	16.7

*Values are means [95% CI]*, *absolute or percent values*.

### Morphokinetics and variables of dynamic monitoring of embryo development

**Fig 1:**
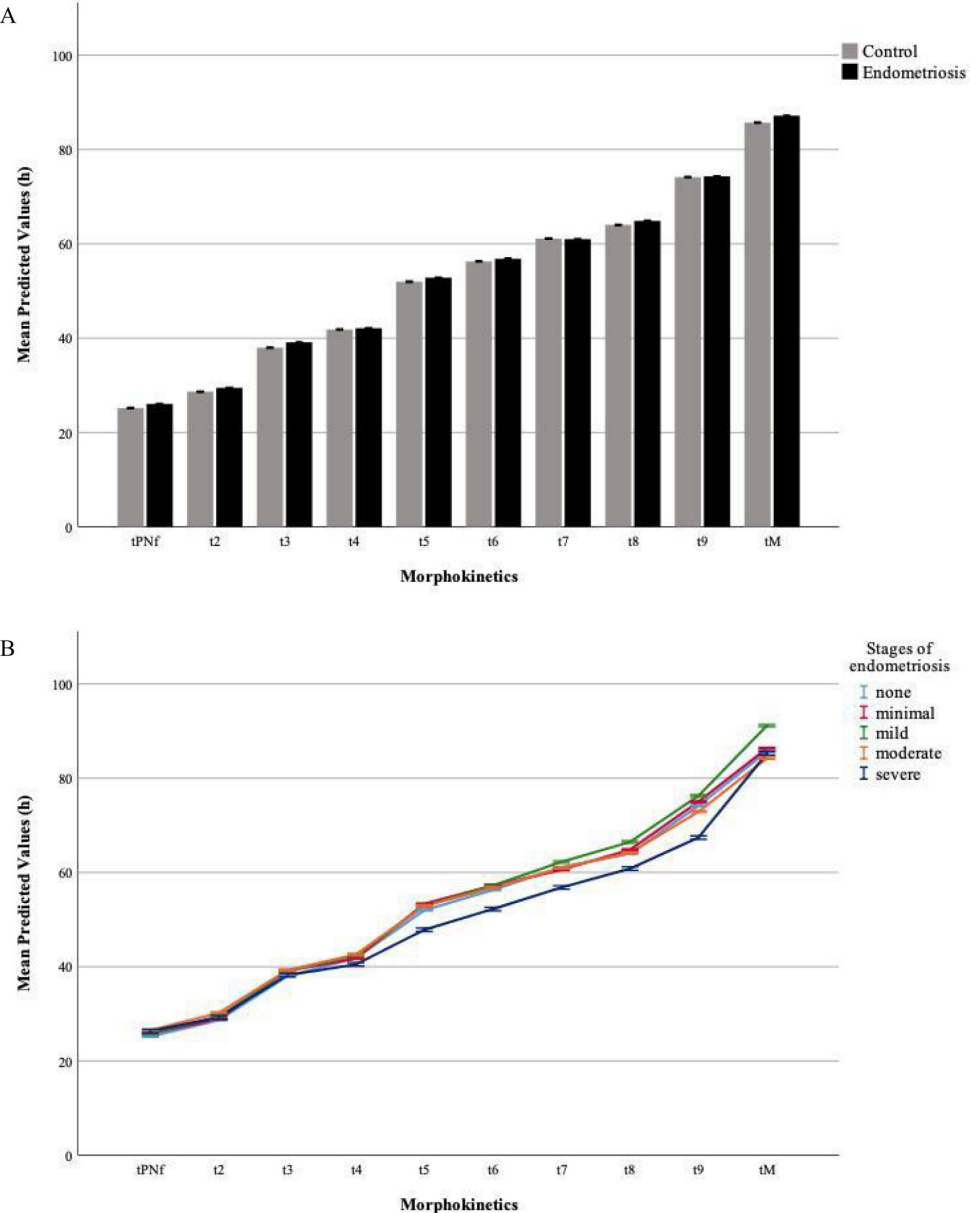
Morphokinetic parameters of early embryo development. A) The mixed-model analysis revealed a significant main effect of time (p<0.001) with post-hoc tests showing that any time needed to reach a specific developmental stage was significantly different from all the others (all p<0.001). Embryos of endometriosis patients showed the same absolute morphokinetic parameters as the control group. B) Subgroup analysis of the different stages of endometriosis and the control group also revealed a significant main effect of time (p<0.001). Looking at the significant overall interaction effect (time*group, p<0.01) more in detail, embryos of severe endometriosis patients reached t9 faster than patients with minimal or moderate endometriosis as well as the control group (all p<0.01). Time gain equalized again at tMor. Data are shown as mean predicted values [95% CI].

Descriptive statistics of the overall data set of morphokinetic parameters in the control and the endometriosis group as well as the different stages of endometriosis can be found in Table 3. Development for all analyzed embryos ranged from tPNf (25.27 ± 3.55 h) to tM (86.78 ± 11.61 h). The mixed-model analysis revealed a significant main effect of time (p<0.001), with post-hoc tests showing that any time needed to reach a specific developmental stage was significantly different from all the others (all p<0.001). Embryos of endometriosis patients showed the same absolute morphokinetic parameters as the control group (Fig 1A).

**Table 3:** Morphokinetic parameters in the control and endometriosis group as well as in the different stages of endometriosis.

MORPHOKINETIC PARAMETERS	CONTROL GROUP	ALL ENDOMETRIOSIS	INDIVIDUAL STAGES OF ENDOMETRIOSIS			
	*N = 596*	*N = 552*	*minimal* *N = 212*	*mild* *N = 143*	*moderate* *N = 154*	*severe* *N = 43*
tPNf	24.9 [24.6, 25.2]	25.7 [25.4, 26.0]	25.7 [25.2, 26.3]	25.8 [25.1, 26.4]	25.6 [25.0, 26.1]	25.6 [24.5, 26.7]
t2	28.5 [28.1, 29.0]	29.4 [29.0, 29.9]	29.0 [28.4, 29.6]	29.4 [28.5, 30.3]	29.8 [28.8, 30.8]	29.7 [28.3, 31.1]
t3	37.9 [37.3, 38.5]	39.1 [38.5, 39.6]	39.3 [38.5, 40.1]	39.1 [37.9, 40.2]	38.9 [37.8, 39.9]	38.6 [36.9, 40.2]
t4	41.5 [40.9, 42.2]	41.8 [41.3, 42.4]	41.7 [40.8, 42.6]	42.0 [40.8, 43.2]	42.1 [40.9, 43.3]	40.9 [39.3, 42.5]
t5	51.3 [50.4, 52.3]	52.5 [51.6, 53.3]	53.5 [52.1, 55.0]	52.5 [50.6, 54.3]	51.7 [50.2, 53.2]	49.9 [47.2, 52.7]
t6	55.4 [54.6, 56.3]	56.1 [55.3, 57.0]	57.1 [57.7, 58.4]	56.3 [54.5, 58.1]	55.1 [53.8, 56.5]	54.4 [51.5, 57.4]]
t7	59.8 [58.8, 60.7]	60.0 [59.1, 61.0]	59.6 [58.0, 61.2]	61.8 [59.8, 63.7]	59.0 [57.3, 60.6]	59.7 [56.3,63.2]
t8	62.6 [61.5, 63.7]	63.7 [62.6, 64.9]	63.8 [61.9, 65.7]	65.6 [63.1, 68.0]	62.2 [60.3, 64.2]	61.4 [58.6, 64.3]
t9	72.8 [71.3, 74.4]	74.2 [72.7, 75.8]	75.5 [72.7, 78.2]	75.7 [72.7, 78.7]	72.3 [69.1, 75.5]	71.1 [67.1, 75.1]
tMor	86.1 [84.6, 87.6]	87.4 [86.1, 88.7]	86.7 [84.8, 88.6]	91.2 [88.3, 94.1]	84.6 [81.8, 87.4]	88.0 [84.6, 91.5]

Time in hours are presented as means [95% CI]; tPNf (time of pronuclei disappearance); t2-t9 (two to nine discrete cells); tMor (end of compaction process, last frame before cavity formation)

Subgroup analysis of the different stages of endometriosis and the control group revealed the same main effect of time (p<0.001), an overall main effect of group (p<0.05) and an overall significant interaction effect (time*group, p<0.01). Looking at Bonferroni-corrected group comparisons, however, no significant differences were visible. Analyzing the interaction effects (Fig 1B), embryos from patients with severe endometriosis showed significant temporal timing alterations in comparison to the other groups at t9, reaching this stage faster than patients with minimal and moderate endometriosis as well as the control group (all p<0.01). Time gain equalized again at tMor. It has to be noted that embryos from patients with severe endometriosis reached tPNf slower than patients with mild endometriosis as well as t5, t6, and t7 faster than patients with moderate endometriosis. After correcting for multiple comparisons, however, these significances vanished and only the significant difference of the interaction effects at t9 remained (severe vs. moderate, p<0.05; severe vs. minimal and vs. control, both p<0.01).

**Fig 2:**
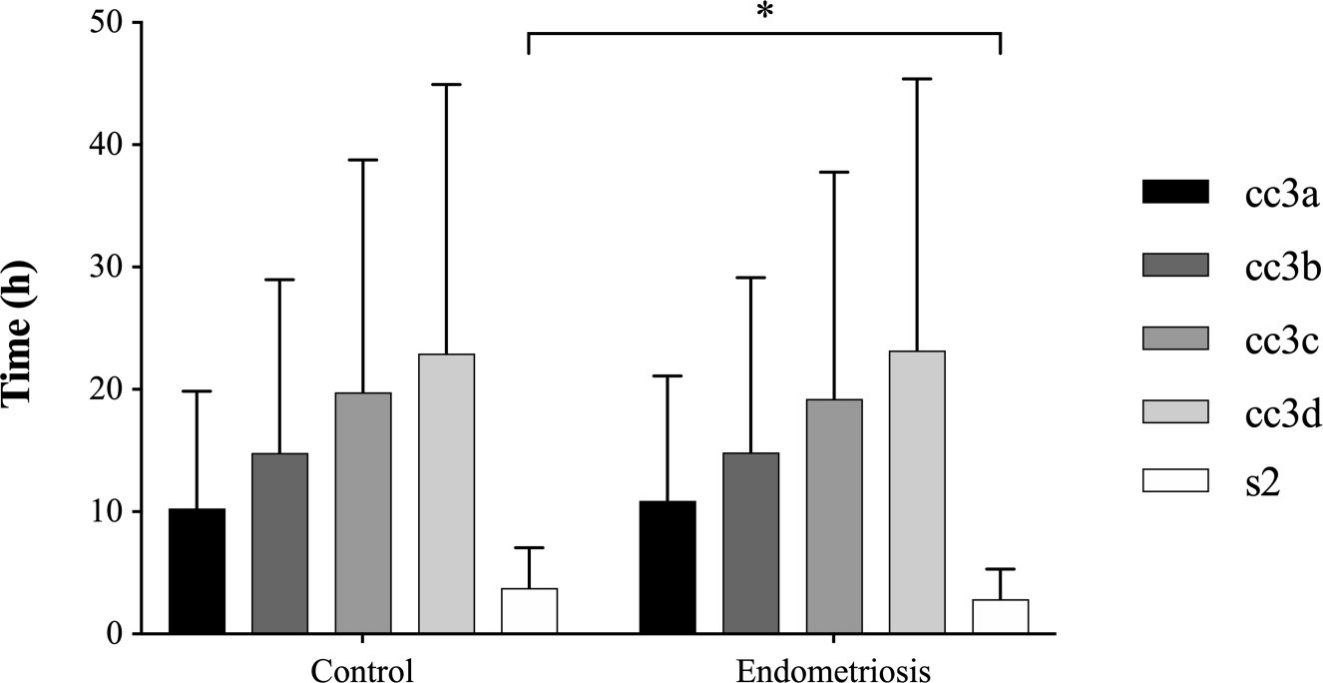
Variables of dynamic embryo development. Synchronization of cell divisions (s2) was significantly lower in the endometriosis than in the control group. Significant differences are indicated as follows: *p<0.05; Data are shown as mean values [95% CI].

Variables of dynamic monitoring of embryo development did not significantly differ between endometriosis and control group except for the synchronity of the two blastomere divisions within the second cell cycle (s2) being significantly faster in the endometriosis group (p<0.05, Fig 2). Subgroup analysis of the different stages of endometriosis, however, did not reveal any significant differences between groups for ECC2, ECC3 or s3.

### Embryo cleavage pattern and outcome analysis

Embryo analysis for abnormal cleavage pattern revealed 17 cases (3.1%) of direct and 2 cases (0.4%) of reverse cleavage embryos in the endometriosis group compared to 17 cases (2.9%) of direct and 4 cases (0.7%) of reverse cleavage in the control group. There was no significant association between embryo cleavage and having/not having endometriosis- also when looking at the different stages of the disease.

Looking only at transferred embryos, there was no overall relationship between fetal heartbeat or live birth rate and the presence or not of endometriosis. Out of 131 transferred embryos in the control group, 33 (25.2%) had a fetal heartbeat and 32 (24.4%) led to live birth, while out of 119 transferred embryos in the endometriosis group, 30 (25.2%) and 27 (22.7%) had a fetal heartbeat and led to live birth, respectively. Looking at fetal heartbeat and live birth rate across the different stages of endometriosis, no association could be found (minimal: 33.3% and 28.2%, mild: 21.2% and 21.2%, moderate: 22.5% and 22.5%, severe: 14.3% and 0%, respectively). The day of embryo transfer did not show any overall significant relationship with endometriosis (χ^2^ (2, n = 236) = 1.18, p = 0.56), fetal heartbeat (χ^2^ (2, n = 236) = 4.95, p = 0.084) or live birth rate (χ^2^ (2, n = 236) = 3.58, p = 0.17). Only looking at the endometriosis group, however, the lowest fetal heartbeat rate was observed for embryos transferred at day 4 (p<0.05). In detail, fetal heartbeat rates were of 31.2%, 5.0% and 28.6% respectively for embryos transferred at day 3, 4 and 5. However, when looking individually at the different stages of endometriosis, none of the associations (day of transfer related to fetal heartbeat or live birth rate) were significant.

In addition, overall, fetal heartbeat and live births could be rather monitored in embryos that reached t2 faster (both r = 0.14, p<0.05, n = 250). Separately investigating the control and endometriosis group, however, these associations vanished in the control group and only remained in the endometriosis group for fetal heartbeat and t2 (r = 0.18, p<0.05, n = 119). Nonetheless, it has to be noted that, although significant, these associations can be considered weak and results should be taken with caution.

## Discussion

In the present study we showed that endometriosis accelerates synchronization of early embryo cell divisions within the second cell cycle, but does not change absolute morphokinetic variables in endometriosis patients. There was no evidence that endometriosis affected the (clinical) outcome of ART-treatment in terms of average number of cycles, transferred embryos/cycle, implanted embryos/cycle, fetal heartbeat or live birth rate. On a subgroup level we observed that embryos from severe endomtriosis patients reached t9 faster than the other groups. To the best of our knowledge, this is the first study investigating embryo morphokinetics, fetal heart beat and live birth rate, in relation to endometriosis and also taking into consideration the differences between the different ASRM stages of endometriosis and the control group.

The assessment of morphokinetics in early embryo development has been shown to be valuable as additional selection criterion in ART-treatment. The idea to unify and standardize morphokinetic variables was introduced by Ciray and coworkers in 2014 to understand and examine the optimal morphokinetic embryo profile [23]. Aside from absolute and dynamic monitoring parameters, relative kinetic expression patterns were proposed as additional information for the assessment of good embryo quality [26]. All of these calculations are consistently based on absolute monitoring parameters. Only two studies evaluated the effect of endometriosis on morphokinetics of the developing embryo [15,16]. The authors suggested descreased cleavage synchronicity from 2- to 8-cell and increased cleavage synchronicity from 4- to 8-cell stage [15]. The duration of the first cell cycle (ECC1) appeared to be faster compared to the one of the control group [16]. In addition, a deceleration of 3- to 4-cell stage (s2) in patients suffering from endometriosis was found [16], possibly due to insufficient statistical analysis. However, both studies did not observe any differences in absolute morphokinetic variables between endometriosis and control group, which is in line with our results. Interestingely, in our study the synchronicity of the two blastomere divisions within the second cell cycle (s2) was found to be significantly accelerated in the endometriosis group compared to the control group. This increase in synchronization may be caused by the inflammatory environment prevailing and contributing to the pathophysiology of endometriosis [27]. Furthermore, cellular rearrangement processes and DNA repair mechanisms caused by oxidative stress may also be a possible explanation for the observed alteration in the cell division process.

It is commonly known that endometriosis exhibits harmful effects on oocyte quality [28], which is one of the major causes in the pathomechanism of the disease and could cause alterations of cleavage synchronicity [15,16]. Furthermore, patients who received embryos derived from endometriotic ovaries showed reduced implantation rates [29]. Meta-analysis, however, suggested no relevant difference in the chance of achieving pregnancy and live birth in endometriosis versus control patients [7]. Although our study design was only powered to thoroughly investigate embryo morphokinetics and did not focus on clinical or ART-treatment outcome parameters, our results suggest no differences between groups regarding retrieved, transferred or implanted embryos/cycle, fetal heart beat or live birth rate. However, we want to emphasize that these results should be interpreted with caution.

Interestingly, we found weak but, nevertheless, significant correlations between fetal heartbeat and the morphokinetic parameter t2. Although it is important to take these results with caution, one could speculate that reaching two discrete cells as soon as possible in order to start “correct” embryo development is crucial. This would suggest t2 to potentially be a marker for good embryo quality in endometriosis patients.

Early cleavage has already been described to result in a significantly higher rate of good quality embryos with elevated implantation rates compared to late-cleaving embryos [30], which is in line with our findings. These results make it tempting to speculate that slight disease-specific morphokinetic differences in the synchronization of cell divisions could possibly influence the overall outcome of ART-treatment and could help to explain controversial results in literature.

In addition, a deeper look into the embryo cleavage pattern revealed no significant association between embryo cleavage (normal, direct or reverse cleavage) and the presence of endometriosis, assuming that differences in synchronization result from different circumstances rather than endometriosis and that the disease does not favor the development of abnormal cleavage pattern in the embryo.

In recent publications dealing with embryo morphokinetics, endometriosis has been analyzed as one distinct group not taking the different stages of the disease into account [15,16]. Hence, we performed an exploratory subgroup analysis of the 4 different stages of endometriosis. Interestingly, this evaluation revealed deviations of the severe endometriosis group from the minimal and moderate endometriosis group as well as the control group by reaching t9 faster. Development in the severe endometriosis group was delayed at tMor, where time gain equalized between groups. Therefore, at first glance, embryo development appeared similar among groups and deviations for severe endometriosis patients were only revealed by further exploratory analysis. These results may represent important missing information that was not yet taken into account by previous studies—especially in patients suffering from the most severe stage of endometriosis [7,31]—but they are unlikely to ultimately explain ART-treatment outcome. In addition, due to the relatively low number of cases, especially in the severe group, and different sample sizes in the four endometriosis groups the mixed-model results of the morphokinetic analysis must be interpreted with caution. However, post-hoc power analysis showed sufficient statistical power (1-beta>0.8) to have had detected any significant difference between groups already with a total sample size of n = 130 (n = 26 embryos/group). Nonetheless, results should be confirmed with a higher sample size in future investigations.

The question of whether the day of embryo transfer should be modified in patients suffering from endometriosis is still under debate. A current meta-analysis showed no differences in reproductive outcomes comparing blastocyst (d5-d6) and cleavage-stage (d2-d3) embryo transfer in clinical practice [32]. In this context, we investigated the association between the day of embryo transfer and presence of endometriosis as well as the individual stages of the disease. Our findings confirm the data found in the meta-analysis for both endometriosis and its individual stages, implicating that there is no need for a change of general transfer policy in patients suffering from the disease.

Retrospective data analysis and the selected control group could be seen as study limitations. In our opinion, patients with unexplained female infertility represent the most suitable control since they are theoretically in a predominantly healthy condition without disease-specific impairments such as tubal factor. The big heterogeneity in study designs and eligibility criteria for endometriosis and control groups in literature makes a general consensus even more complex. Hence, future studies should try to unify their inclusion criteria in order to make presented results more comprehensible and trustworthy.

In conclusion, the current study showed that endometriosis accelerates synchronization of the two blastomere divisions within the second cell cycle, but does not change absolute morphokinetic parameters in endometriosis patients. Disease-induced changes in this synchronization pattern might be a missing explanation for contradicting results in literature regarding the impairments in ART-treatment outcome in endometriosis patients. These findings demonstrate once again the complexity of the endometriotic pathomechanism and require additional research to further unravel the black box of endometriotic development.

## Supporting information

S1 Table Dataset. (DOCX)
