# Supplementary material for: Endometriosis accelerates synchronization of early embryo cell divisions but does not change morphokinetic dynamics in endometriosis patients
Source: PLoS One. 2019 Aug 1;14(8):e0220529. doi: 10.1371/journal.pone.0220529 (PMC6675061; doi:10.1371/journal.pone.0220529)
Supplement: S1 Table — (DOCX) [file pone.0220529.s001.docx]

| Fig # | **Mean (h)** | **SD (h)** | **Statistical Method used** | **p value** | **# samples** |
| --- | --- | --- | --- | --- | --- |
| **Fig 1A** |  |  | Linear mixed effect model (restricted maximum likelihood approach, RML) approach, including two fixed factors (repeated: developmental stages tPNf-tM, group effect: endometriosis yes/no) with Bonferroni post-hoc comparisons, a random intercept (patient ID) and a random slope as well as three covariates (age, BMI, cycle). Interaction effects were also post-hoc corrected for multiple testing (Bonferroni). | Repeated effect: ***p<0.001  Group effect: p>0.05  Interaction effect:  p>0.05 |  |
| tPNf (Con) | 24.97 | 3.51 |  |  | 523 |
| tPNf (Endo) | 25.84 | 3.69 |  |  | 444 |
| t2 (Con) | 28.66 | 5.95 |  |  | 541 |
| t2 (Endo) | 29.45 | 5.51 |  |  | 465 |
| t3 (Con) | 37.98 | 7.45 |  |  | 538 |
| t3 (Endo) | 39.12 | 6.52 |  |  | 465 |
| t4 (Con) | 41.70 | 8.39 |  |  | 522 |
| t4 (Endo) | 42.04 | 6.76 |  |  | 449 |
| t5 (Con) | 51.55 | 11.56 |  |  | 495 |
| t5 (Endo) | 52.58 | 9.68 |  |  | 414 |
| t6 (Con) | 55.55 | 10.43 |  |  | 459 |
| t6 (Endo) | 56.23 | 8.78 |  |  | 385 |
| t7 (Con) | 59.79 | 10.48 |  |  | 398 |
| t7 (Endo) | 59.97 | 9.17 |  |  | 325 |
| t8 (Con) | 62.72 | 11.10 |  |  | 359 |
| t8 (Endo) | 63.86 | 10.74 |  |  | 293 |
| t9 (Con) | 72.66 | 11.66 |  |  | 200 |
| t9 (Endo) | 74.26 | 11.02 |  |  | 166 |
| tM (Con) | 85.95 | 12.33 |  |  | 245 |
| tM (Endo) | 87.71 | 10.66 |  |  | 204 |
| **Fig 1B** |  |  |  |  |  |
| tPNf (Con) | 24.97 | 3.51 | Linear mixed effect model (RML) approach, including two fixed factors (repeated: developmental stages tPNf-tM, group effect: stages of endometriosis) with Bonferroni post-hoc comparisons, a random intercept (patient ID) and a random slope as well as three covariates (age, BMI, cycle). Interaction effects were also post-hoc corrected for multiple testing (Bonferroni). | Repeated effect: ***p<0.001  Group effect: p>0.05  Interaction effect:  p<0.05 | 523 |
| tPNf (minimal) | 25.92 | 3.72 |  |  | 167 |
| tPNf (mild) | 25.83 | 3.96 |  |  | 128 |
| tPNf (moderate) | 25.97 | 3.48 |  |  | 125 |
| tPNf (severe) | 25.53 | 3.16 |  |  | 24 |
| t2 (Con) | 28.66 | 5.95 |  |  | 541 |
| t2 (minimal) | 28.89 | 4.42 |  |  | 171 |
| t2 (mild) | 29.44 | 5.78 |  |  | 130 |
| t2 (moderate) | 30.19 | 6.43 |  |  | 138 |
| t2 (severe) | 29.26 | 5.08 |  |  | 26 |
| t3 (Con) | 37.98 | 7.45 |  |  | 538 |
| t3 (minimal) | 39.17 | 6.24 |  |  | 171 |
| t3 (mild) | 39.09 | 6.88 |  |  | 130 |
| t3 (moderate) | 39.25 | 6.80 |  |  | 138 |
| t3 (severe) | 38.21 | 5.22 |  |  | 26 |
| t4 (Con) | 41.70 | 8.39 |  |  | 522 |
| t4 (minimal) | 41.58 | 6.03 |  |  | 163 |
| t4 (mild) | 42.30 | 7.15 |  |  | 127 |
| t4 (moderate) | 42.64 | 7.57 |  |  | 134 |
| t4 (severe) | 40.42 | 4.07 |  |  | 25 |
| t5 (Con) | 51.55 | 11.56 |  |  | 495 |
| t5 (minimal) | 53.12 | 9.87 |  |  | 155 |
| t5 (mild) | 52.84 | 10.37 |  |  | 117 |
| t5 (moderate) | 52.43 | 8.99 |  |  | 122 |
| t5 (severe) | 47.79 | 7.06 |  |  | 20 |
| t6 (Con) | 55.55 | 10.43 |  |  | 459 |
| t6 (minimal) | 56.75 | 8.59 |  |  | 142 |
| t6 (mild) | 56.50 | 10.12 |  |  | 112 |
| t6 (moderate) | 55.93 | 7.68 |  |  | 111 |
| t6 (severe) | 52.19 | 6.92 |  |  | 20 |
| t7 (Con) | 59.79 | 10.48 |  |  | 398 |
| t7 (minimal) | 58.57 | 8.64 |  |  | 109 |
| t7 (mild) | 61.83 | 10.44 |  |  | 101 |
| t7 (moderate) | 59.96 | 8.61 |  |  | 98 |
| t7 (severe) | 57.93 | 5.40 |  |  | 17 |
| t8 (Con) | 62.72 | 11.10 |  |  | 359 |
| t8 (minimal) | 62.92 | 10.21 |  |  | 102 |
| t8 (mild) | 65.72 | 12.45 |  |  | 92 |
| t8 (moderate) | 63.14 | 9.79 |  |  | 84 |
| t8 (severe) | 62.87 | 6.56 |  |  | 15 |
| t9 (Con) | 72.66 | 11.66 |  |  | 200 |
| t9 (minimal) | 74.55 | 10.26 |  |  | 58 |
| t9 (mild) | 75.47 | 11.11 |  |  | 52 |
| t9 (moderate) | 73.31 | 12.07 |  |  | 47 |
| t9 (severe) | 70.31 | 9.85 |  |  | 9 |
| tM (Con) | 85.95 | 12.33 |  |  | 245 |
| tM (minimal) | 86.83 | 9.14 |  |  | 80 |
| tM (mild) | 91.29 | 11.54 |  |  | 58 |
| tM (moderate) | 85.66 | 11.30 |  |  | 50 |
| tM (severe) | 85.57 | 10.12 |  |  | 16 |
| **Fig 2** |  |  |  |  |  |
| cc3a (Con) | 10.27 | 7.64 | Linear mixed effect model (RML) with one fixed factor (endometriosis yes/no) and one random factor (patient ID) with Bonferroni post-hoc comparisons including age, BMI and cycle as covariates. | p>0.05 | 495 |
| cc3a (Endo) | 10.80 | 7.10 |  |  | 415 |
| cc3b(Con) | 14.77 | 7.25 |  | p>0.05 | 459 |
| cc3b (Endo) | 14.88 | 6.13 |  |  | 386 |
| cc3c(Con) | 19.71 | 8.46 |  | p>0.05 | 398 |
| cc3c (Endo) | 19.22 | 7.20 |  |  | 326 |
| cc3d(Con) | 22.91 | 9.35 |  | p>0.05 | 359 |
| cc3d (Endo) | 23.28 | 9.12 |  |  | 293 |
| s2 (Con) | 3.90 | 5.89 |  | p<0.05 | 522 |
| s2 (Endo) | 3.00 | 4.83 |  |  | 449 |
| Values are presented as mean and standard deviation (SD); tPNf (time of pronuclei disappearance); t2-t9 (two to nine discrete cells); tMor (end of compaction process, last frame before cavity formation); Con, Control; Endo, Endometriosis; | | | | | |
